# A *Yersinia ruckeri* TIR Domain-Containing Protein (STIR-2) Mediates Immune Evasion by Targeting the MyD88 Adaptor

**DOI:** 10.3390/ijms20184409

**Published:** 2019-09-07

**Authors:** Tao Liu, Wen-Yan Wei, Kai-Yu Wang, Er-Long Wang, Qian Yang

**Affiliations:** 1 Department of Basic Veterinary, Veterinary Medicine College, Sichuan Agricultural University, Chengdu 611134, China; 2 Institute of Fisheries of Chengdu Agriculture and Forestry Academy, Chengdu 611130, China; 3 Key Laboratory of Animal Disease and Human Health of Sichuan Province, Chengdu 611130, China

**Keywords:** *Yersinia ruckeri*, TIR, STIR-2, MyD88, immune evasion

## Abstract

TIR domain-containing proteins are essential for bacterial pathogens to subvert host defenses. This study describes a fish pathogen, *Yersinia ruckeri* SC09 strain, with a novel TIR domain-containing protein (STIR-2) that affects Toll-like receptor (TLR) function. STIR-2 was identified in *Y. ruckeri* by bioinformatics analysis. The toxic effects of this gene on fish were determined by in vivo challenge experiments in knockout mutants and complement mutants of the *stir-2* gene. In vitro, STIR-2 downregulated the expression and secretion of IL-6, IL-1β, and TNF-α. Furthermore, the results of NF-κB-dependent luciferase reporter system, co-immunoprecipitation, GST pull-down assays, and yeast two-hybrid assay indicated that STIR-2 inhibited the TLR signaling pathway by interacting with myeloid differentiation factor 88 (MyD88). In addition, STIR-2 promoted the intracellular survival of pathogenic *Yersinia ruckeri* SC09 strain by binding to the TIR adaptor protein MyD88 and inhibiting the pre-inflammatory signal of immune cells. These results showed that STIR-2 increased virulence in *Y. ruckeri* and suppressed the innate immune response by inhibiting TLR and MyD88-mediated signaling, serving as a novel strategy for innate immune evasion.

## 1. Introduction

Virulence factors are extracellular proteins produced by pathogens to establish and maintain disease. Most virulence factors are enzymes that promote the growth and development of pathogens. For instance, *Streptococcus* [1], *Staphylococcus* [2], and *Clostridium* [3] produce hyaluronidase, which promotes microbial growth in tissues by hydrolyzing hyaluronic acid. *Streptococcus* and *Staphylococcus* produce large amounts of proteolytic enzymes [4], nucleases [5], and lipases [6] that depolymerize host proteins, nucleic acids, and fats, respectively. However, usually, the host interacts with extracellular proteins to reduce their likelihood of causing disease [7,8]. In addition, the expression of bacterial virulence factors varies among bacterial species, and these factors may have distinct effects in different strains of the same species. In addition to virulence factors, genes involved in bacterial viability are a cause for concern because they help bacteria adapt to several living environments (including survival in the host and other cells). The contribution of force may be more stable and lasting.

*Yersinia ruckeri* is the etiological agent of enteric redmouth disease (ERM) and infects several trout species worldwide [9]. Over the past few decades, ERM has caused substantial economic losses in fish from veterinary costs and mortality [10]. Despite the administration of an effective vaccine to fish, outbreaks continue to occur and are caused by specific bacterial strains [11,12]. However, little is known about the virulence mechanisms of *Y. ruckeri*. Many potential virulence factors have been reported, including extracellular toxins [13,14], a high-affinity iron uptake system designated ruckerbactin [13], flagellar motility genes [15], and a two-component system [16]. To further understand *Y. ruckeri* pathogenicity, we previously sequenced the complete genome of *Y. ruckeri* SC09, which is a highly virulent strain isolated in China from fish with severe septicemia [17]. The genome of the SC09 strain contains several horizontal gene transfer (HGT) events, and many genes involved in immune evasion are transferred horizontally, which prompted us to further study the correlation between immune evasion and the virulence of *Y. ruckeri*.

The most representative horizontal transfer elements in the genome of the SC09 strain are integrative and conjugative elements (ICEs). ICEs are a class of newly recognized mobile DNA elements in prokaryotes [18]. Many ICEs harbor a tyrosine recombinase gene and are flanked by direct repeats corresponding to the 3′ end of a conserved gene (e.g., a tRNA gene), which suggests genomic integration in the 3′ end of this gene via site-specific recombination [19]. Furthermore, ICEs typically have a core of conserved modular structures that mediate their integration, excision, conjugation, and regulation, and this core region is interspersed with accessory regions that are variably present across members of a species [20]. A mobile element designated ICE(r2) (NJ56_RS12425-NJ56_RS12600) in the SC09 strain is integrated between an intact or partial tRNA-Asn copy. Moreover, a class of bacterial proteins homologous to the Toll/IL-1 receptor (TIR) domain was identified; these proteins were encoded by ICE(r2) and acted as accessory genes. One of these proteins, STIR-2, which is encoded by NJ56_RS12445, is present in SC09 but absent in other *Y. ruckeri* strains, including RS41 (NCBI RefSeq: NZ_CQBN00000000.1), OMBL4 (NCBI RefSeq: NZ_CPUZ00000000.1), and CSF007-82 (NCBI RefSeq: NZ_CCYO00000000.1). The TIR domain is essential for the interaction between the Toll-like receptor (TLR) and its adaptor. Binding of the TIR domain to the corresponding ligand initiates intracellular signal transduction and mediates the production of various inflammatory factors by activating the nuclear translocation of the transcription factor NF-κB [21]. In infections, distinct TLRs recognize different bacterial structures. TLR2 and TLR4 recognize and bind to the phosphatidic acid and LPS in the bacteria wall, respectively [22]. Accordingly, is has been shown that a TIR-containing protein in the SC09 strain, STIR-2, targets TLR signaling to facilitate escape from the host innate immune response and enhance virulence. In fact, previous studies detected the intracellular survival of *Y. ruckeri* [23].

In the present study, a novel *Y. ruckeri* STIR-2 was identified through in vivo and in vitro experiments. The results showed that STIR-2 efficiently inhibited TLR signaling and contributed to toxicity in vitro and in vivo. Moreover, we propose that STIR-2 represents a new class of virulence factors that modulate host inflammatory response during infection with *Y. ruckeri*.

## 2. Results

### 2.1. *Genomic Location of Stir-2 in* Yersinia ruckeri

**Figure 1:**
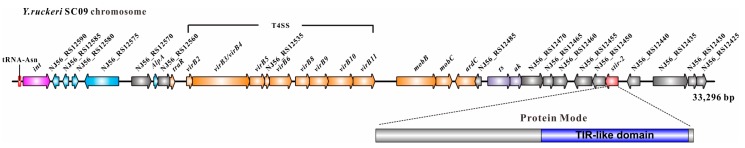
ICE(r2) with putative ICE regions in bacterial genomes. Operon structure of integrative and conjugative elements (ICEs). Int, integrase; T4SS, type 4 secretory system.

Among prokaryotic microorganisms, integrative and conjugative elements (ICEs) are a large class of DNA horizontal transfer elements [24]. Almost all ICEs contain a tyrosine recombinase gene, which is often flanked by a conserved gene by direct repeats [25]. ICEs typically have some modular structures that mediate their integration, excision, conjugation, and regulation, and these structures are interspersed with accessory regions that are variably present across different species [26]. The mobile element ICE(r2) (NJ56_RS12425-NJ56_RS12600) in the SC09 strain was integrated between an intact or partial tRNA-Asn copy. ICE(r2) is flanked on one side by an integrase gene and carries a VirB/VirD4 type IV secretion system operon (T4SS, NJ56_RS12510-NJ56_RS12550) that may mediate conjugation from donor to recipient cells analogous to conjugative plasmid translocation. A bacterial protein homologous to the Toll/IL-1 receptor (TIR) domain encoded by SC09-ICE(r2), which acts as an accessory gene, has also been identified. This protein, designated STIR-2, is encoded by NJ56_RS12465 (Figure 1, genes are colored in red). The binding of the TIR domain to the corresponding ligand (such as MyD88) activates intracellular signal transduction and mediates the production of inflammatory factors by nuclear translocation of the transcription factor NF-κB [27]. TIR domain interactions play a pivotal role in mediating innate immunity and identifying pathogens [28]. Therefore, it is reasonable to hypothesize that TIR domain-containing proteins (Tcps) from *Y. ruckeri* SC09 disrupt the TLR signal transduction system in host cells to facilitate escape from the host innate immune and enhance bacterial survival.

### 2.2. STIR-2 is Required for Yersinia ruckeri SC09 Infection in Vivo and in Vitro

**Figure 2:**
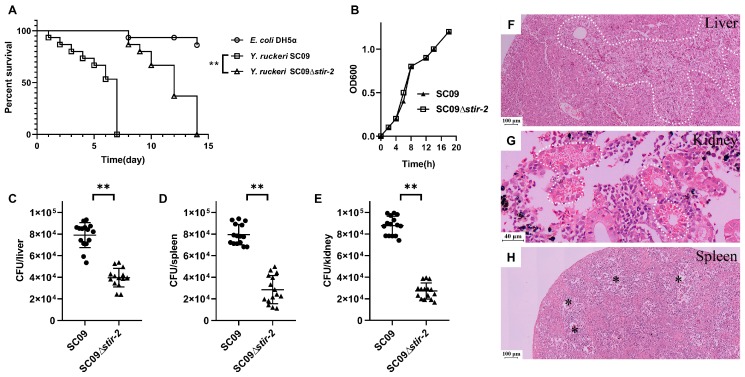
STIR-2 is required for *Yersinia ruckeri* SC09 infection in vivo. (**A**) Rainbow trout survival curve. Rainbow trout were infected with either wild-type *Y. ruckeri* SC09 or SC09Δ*stir-2*. The Mantel-Cox test was used with ** *p* < 0.01. Fish infected with *E. coli* DH5α, which has low toxicity to fish, acted as a negative control. (**B**) Growth curve of wild-type *Y. ruckeri* SC09 and SC09Δ*stir-2*. (**C**–**E**) Rainbow trout were infected with either *Y. ruckeri* SC09 or SC09Δ*stir-2* (*n* = 15/group). Bacterial load in the liver (**C**), spleen (**D**), and kidney (**E**) was assessed in cultured the tissue homogenates. A non-parametric two-tailed *t*-test was carried out with (**C**) ** *p* < 0.01, (**D**) ** *p* < 0.01, and (**E**) ** *p* < 0.01. (**F**–**H**) Pathological lesions of rainbow trout infected with wild-type *Y. ruckeri* SC09. Necrotic areas in the liver (**F**, white dotted line); glass-like substrate in kidney tubules (**G**, white dotted line); edema in spleen medulla (**H**, asterisk)**.**

To analyze the virulence of *Yersinia ruckeri*, we constructed an in-frame *stir-2*-deletion mutant of SC09 and a ∆*stir-2* + pSTIR-2 mutant complemented with a plasmid containing the *stir-2* gene controlled by its promoter. An in vivo acute infection model of rainbow trout was used to evaluate the involvement of STIR-2 in *Y. ruckeri*-induced disease in fish. The wild-type SC09 strain caused 50% lethality 6 days after inoculation (Figure 2A). In contrast, the SC09Δ*stir-2* mutant slightly but significantly (** *p* < 0.01) attenuated virulence in fish (Figure 2A). Fish infected with *E. coli* DH5α, which has low toxicity to fish, acted as a negative control (Figure 2A). The differences in toxicity were not due to an in vitro growth defect of the mutant (Figure 2B). SC09Δ*stir-2*-infected fish showed decreased cell recruitment and enhanced bacterial clearance in the liver, spleen, and kidney compared to the wild-type strain (Figure 2C–E, ** *p* < 0.01). Tissue damage was detected in fish infected with wild-type SC09 but not with SC09Δ*stir-2*. Histopathological examination showed marked changes in the liver, kidney, and spleen of infected fish (Figure 2E–G). Severe vacuolar degeneration and necrosis of hepatic cells were observed in the liver (Figure 2F); glass-like substrate was observed in kidney tubules (Figure 2G); the spleen medulla became loose and edematous (Figure 2H). These results showed that STIR-2 was required for *Y. ruckeri* SC09 infection in vivo.

**Figure 3:**
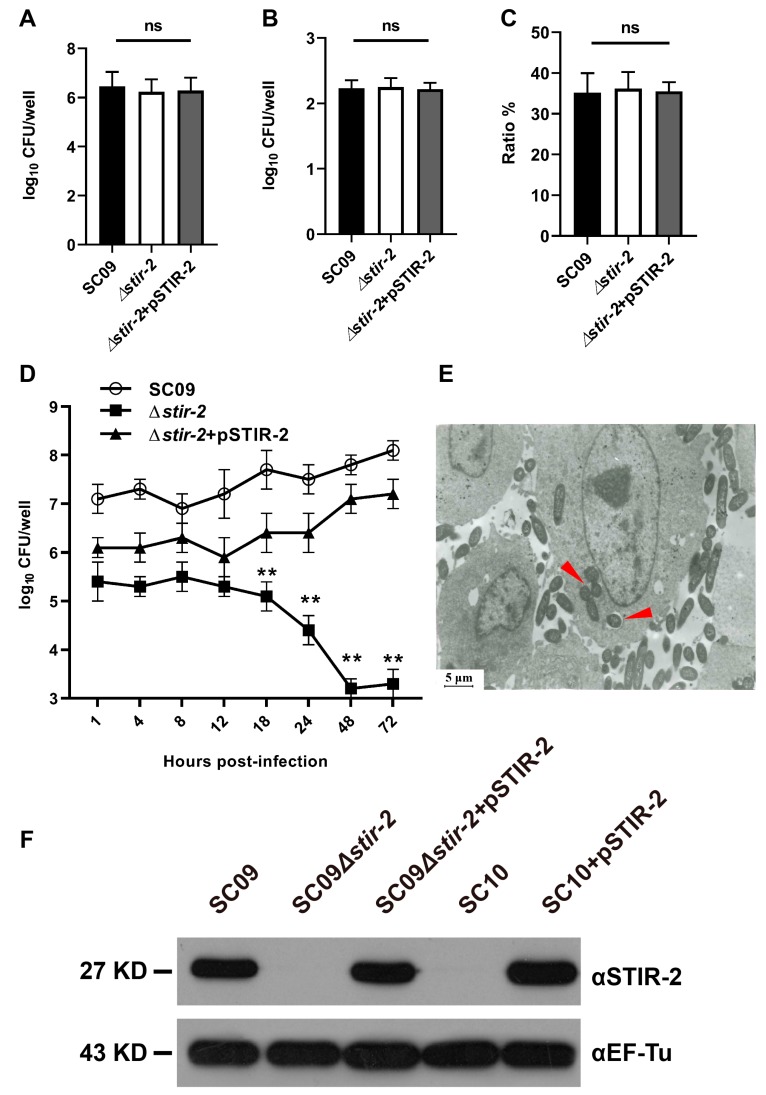
The knockout of the *stir-2* gene in the *Yersinia ruckeri* SC09 strain affects intracellular bacterial survival but not bacterial adherence and invasion. Primary rainbow trout head kidney macrophages infected with the wild-type *Y. ruckeri* SC09 strains, *Y. ruckeri* SC09Δ*stir-2* mutant, or *Y. ruckeri*∆*stir-2* + pSTIR-2 mutant at a MOI of 1.5. There were no significant changes in bacterial adherence (**A**), invasion (**B)**, and invasion ratios (number of intracellular bacteria/number of adherent bacteria) (**C**). ns, the means were not significantly different. (**D**) At 18, 24, 48, and 72 hpi (MOI = 1.5), the intracellular survival of the SC09Δ*stir-2* mutant was significantly lower than that of the wild-type SC09 strain and ∆*stir-2* + pSTIR-2 mutant. ** *p* < 0.01. (**E**) Transmission electron micrographs of primary rainbow trout head kidney macrophages infected the wild-type *Y. ruckeri* SC09 strain (red arrow) at 48 h post infection (hpi). (**F**) Western blot analysis was performed on the bacteria used to infect primary rainbow trout head kidney macrophages. The knockout strain did not express STIR-2 (27 kDa), whereas the wild type strain and the complemented strain expressed STIR-2 (27 kDa). The cytoplasmic protein EF-Tu (43 kDa) was used as a control.

Bacterial adherence, invasion, and intracellular survival were assessed in vitro to confirm whether STIR-2 affects the host innate immune response to enhance bacterial survival. Rainbow trout head kidney macrophages were infected with the wild-type SC09, SC09Δ*stir-2* mutant, or ∆*stir-2* + pSTIR-2 mutant. Bacterial adhesion (Figure 3A), invasion (Figure 3B), and the invasion ratio (number of intracellular bacteria/number of adherent bacteria) (Figure 3C) were not significantly different between the wild-type SC09 and SC09Δ*stir-2* mutant. Therefore, the *stir-2* gene did not appear to enhance the ability of *Y. ruckeri* SC09 to adhere and invade rainbow trout macrophages. However, an intracellular survival assay demonstrated that the survival of the ∆*stir-2* mutant was lower than that of wild-type SC09 and ∆*stir-2* + pSTIR-2 at 18 h post infection (hpi) (Figure 3D), indicating a role of *stir-2* in survival inside rainbow trout macrophages. TEM micrographs showed the presence of intact wild-type SC09 strains inside macrophages at 48 h post-infection and several bacteria inside autophagocytic vacuoles (Figure 3E). In addition, western blot results showed that STIR-2 was not detected in the ∆*stir-2* mutant but was present in the ∆*stir-2* + pSTIR-2 mutant (Figure 3F). The *Y. ruckeri* SC10 strain (lacking *stir-2*) was not detected the STIR-2 protein, but we can express STIR-2 protein in SC10 and detected STIR-2 again (Figure 3F). These results indicated that STIR-2 was required for *Y. ruckeri* SC09 infection in vitro.

### 2.3. STIR-2 Decreases the Secretion and Transcription of Proinflammatory Cytokines

**Figure 4:**
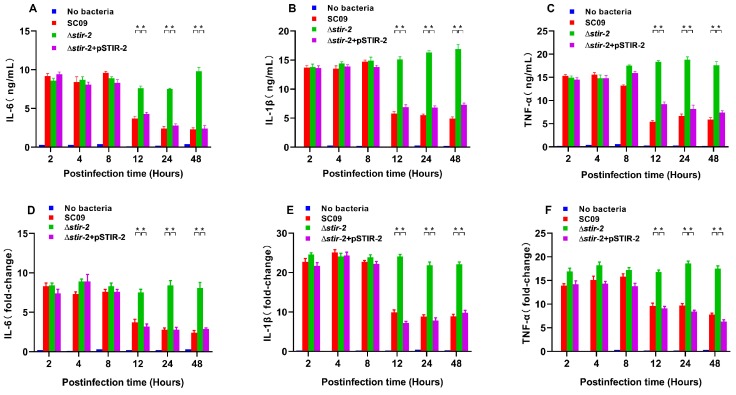
STIR-2 reduces cytokine secretion and expression in macrophages cells. (**A**–**C**) IL-6, IL-1β, and TNF-α secretion in rainbow trout head kidney macrophages either uninfected or infected with the wild-type *Y. ruckeri* SC09 strain, *Y. ruckeri* SC09Δ*stir-2* mutant, or ∆*stir-2* + pSTIR-2 mutant analyzed by ELISA. Cytokine levels were determined at 2, 4, 8, 12, 24, and 48 h after infection. (**D**–**F**) Expression of IL-6, IL-1β, and TNF-α in macrophages either uninfected or infected with the wild-type *Y. ruckeri* SC09 strain, *Y. ruckeri* SC09Δ*stir-2* mutant, or ∆*stir-2* + pSTIR-2 mutant was determined by qPCR. Cytokine expression levels were determined at 2, 4, 8, 12, 24, and 48 h after infection. Error bars indicate the standard deviation of six individual cultures. ** *p* < 0.01, ANOVA on ranks.

An intracellular survival assay was performed in macrophages to assess whether bacterial survival was due to immune evasion or inactivation of innate immunity. To test this hypothesis, the innate response to wild-type SC09, SC09Δ*stir-2* mutant, and ∆*stir-2* + pSTIR-2 mutant was determined in rainbow trout head kidney macrophages. IL-6, IL-1β, and TNF-α levels in culture supernatants were quantified by ELISA. The results showed that infection of macrophages with the ∆*stir-2* mutant increased the expression of IL-6 (Figure 4A), IL-1β (Figure 4B), and TNF-α (Figure 4C) at 18, 24, and 48 hpi compared with macrophages infected with the wild-type SC09 strain or the ∆*stir-2* + pSTIR-2 mutant strain. To further confirm that the effect of *stir-2* on macrophages was due to the decreased transcription of cytokines, we determined the mRNA expression of IL-6, IL-1β, and TNF-α in macrophages. Infection with the ∆*stir-2* mutant increased the expression of IL-6 (Figure 4D), IL-1β (Figure 4E), and TNF-α (Figure 4F) at 18, 24, and 48 hpi, respectively, compared with macrophages infected with the wild-type SC09 strain or the ∆*stir-2* + pSTIR-2 mutant strain. These results indicated that STIR-2 decreased the secretion and transcription of proinflammatory cytokines.

### 2.4. STIR-2 Affects Host TLR Signaling

**Figure 5:**
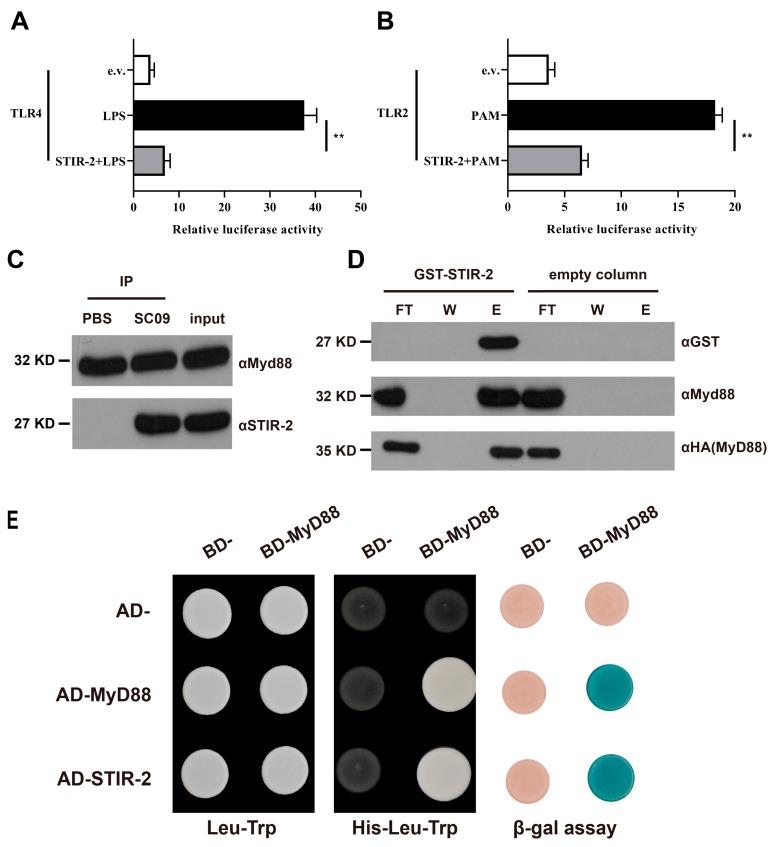
STIR-2 impairs TLR signaling by binding to MyD88. (**A**) The luciferase reporter plasmid and the TLR4 plasmid were transiently transfected into rainbow trout head kidney macrophages with or without the STIR-2 plasmid. After 24 h, the cells were stimulated with LPS for 6 h, and luciferase activity was determined. The white band represents the negative control (empty vector), the black band represents LPS-stimulated cells, and the gray band represents cells transfected with the STIR-2 plasmid and stimulated by LPS. Data correspond to median ± standard errors of the relative luciferase activity from five independent experiments. ** *p* < 0.01. (**B**) This part of the experiment was similar to (**A**), except that the plasmid was TLR2, and the cells were stimulated with PAM. ** *p* < 0.01**.** (**C**) Co-immunoprecipitation (co-IP) experiments were performed on cells infected with wild-type SC09 or PBS (control). After co-IP, the anti-STIR-2 antibody was used to detect protein interactions, and the anti-MyD88 antibody was used to detect proteins bound to beads, and the inputs were detected with anti-MyD88 antibody and anti-STIR-2 antibody, respectively. (**D**) Pull-down experiments were performed by the in vitro expression of HA-MyD88 protein and the prokaryotic expression of GST-STIR-2 protein immobilized on a Ni-NTA resin. An empty vector was used as a control. The anti-HA and anti-MyD88 antibodies were used to detect protein interactions (lower blot) by Western blotting, and the anti-GST antibody was used to detect the binding of the GST-STIR-2 protein to the resin. The flowthrough (FT), two washes (W), and elution (**E**) are shown in each lane. (**E**) Recombinant plasmids containing Gal4 BD and Gal4 AD were co-transformed into yeast cells and screened on plates lacking leucine (Leu) and tryptophan (Trp) (left panel). Gal4 BD- and Gal4 AD-recombinant plasmids were co-transformed into yeast cells and screened on plates lacking histidine (His) in the presence of 20 mM 3AT (middle panel). Transformants grown on this medium present an interaction between STIR-2 and MyD88. The expression of the reporter β-galactosidase gene in yeast produces blue yeast, and indicated the occurrence of protein interactions (right panel). Empty vectors for AD- and BD-plasmids served as negative controls, and MyD88 homodimerization served as a positive control.

Given the results above and the results of bioinformatics analysis (STIR-2 has the potential to disrupt the TLR signaling pathway), an in vitro NF-κB-dependent luciferase reporter system was used to investigate whether STIR-2 affects the TLR signaling pathway. The transient expression of the *stir-2* gene in rainbow trout head kidney macrophages transfected with the NF-κB luciferase reporter vector and the plasmid encoding TLR4 or TLR2 was stimulated by LPS or PAM. STIR-2 inhibited the TLR4-mediated NF-κB response to LPS (Figure 5A). Furthermore, STIR-2 impaired the NF-κB response to the potent TLR2 agonist PAM in TLR2-transfected cells (Figure 5B). These results suggested that STIR-2 may affect key proteins of the TLR signaling pathway, including MyD88. After that, the effect of STIR-2 on MyD88 was assessed. Macrophages were infected with the wild-type SC09 strain and co-immunoprecipitated (CO-IP) to confirm whether STIR-2 and MyD88 belonged to the same complex (Figure 5C). Protein binding was confirmed using purified GST-tagged STIR-2 immobilized on a Ni-NTA resin, which retained HA-MyD88 (Figure 5D). In addition, the results of a yeast two-hybrid assay indicated that STIR-2 bound to MyD88 (Figure 5E).

## 3. Discussion

In the field of medicine and veterinary medicine, researchers are concerned about the consequences of bacterial infection in the host. However, the interaction of most bacteria with the host does not cause significant disease or damage to the host. For most pathogens, asymptomatic colonization or bacterial elimination by the host before causing disease is common during bacteria-host interactions. In medicine, some of the most common pathogens, including *Streptococcus pneumoniae* [29], *Neisseria meningitidis* [30], *Staphylococcus aureus* [31], and *Enterococcus faecalis* [32], can cause severe disease or kill the host. However, from the ratio of exposure/binding rate of these bacteria that cause disease and death are still a minority [33]. In many cases, the asymptomatic presence of bacteria is a prerequisite for disease [34,35,36,37,38]. Therefore, we believe that during the invisible carrying of bacteria in the host, genetic factors that promote bacterial adaptation (including viability and reproductive capacity) and the infection are worthy of attention. Therefore, the discussion about disease and pathogenicity should also consider gene clusters involved in bacterial viability and reproductive capacity. Gene clusters associated with pathogenicity and bacterial survival may be distributed over longer loci, such as genes encoding capsular polysaccharides [39] or lipopolysaccharides [40], or larger polyprotein devices such as the type 3 secretion system (T3SS) [41]. These large loci may also perform horizontal gene transfer. In addition, it is worth considering horizontal transfer elements such as plasmids, phages, integrons, and ICEs [42], which usually carry clusters of disease-associated genes that affect host characteristics and enhance bacterial survival.

The present results indicated that the fish pathogen *Yersinia ruckeri* SC09 produced TLR homologs that inhibited TLR signaling and enhanced immune evasion. We previously performed whole-genome sequencing to obtain the sequence of *Y. ruckeri* SC09 [17] and found specific ICE elements in the bacterial genome by comparative genomics. ICE elements are thought to mediate horizontal gene transfer in bacteria [43]. ICEs can provide the host with specific cargo genes to help the host better adapt to complex environments or provide toxic capabilities to the bacteria [44]. We found that the ICE(r2) element in the *Y. ruckeri* SC09 genome carried a *stir-2* gene (NJ56_RS12465) involved in immune evasion and hypothesized that this gene enhanced bacterial virulence and immune evasion. Moreover, the results of in vivo experiments indicated that the *stir-2* gene was associated with disease in fish. STIR-2 was also shown to increase bacterial burden and tissue damage in fish; *stir-2* from *Y. ruckeri* SC09 promoted the intracellular accumulation of bacteria in vitro; stir-2 can affect the transcription and secretion of proinflammatory cytokines during the infection of primary immune cells of fish. Furthermore, STIR-2 binds to MyD88, resulting in the inhibition of MyD88-induced activation. Therefore, we hypothesize that this gene acts as a virulence factor in bacteria by impairing signal transduction in the innate immune system, helping the bacteria to escape immune surveillance and replicate in host cells. This virulence gene is the first described in *Y. ruckeri* with a role in immune evasion. This finding helps us better understand the virulence of aquatic pathogens and propose more effective methods of prevention and control.

The Toll/interleukin-1 receptor (TIR) domain plays an important role in the innate immune response of animals, including fish [21]. In recent years, the TIR domains of many microorganisms have been identified and found to impair host immunity [28,45]. Tcps are also ubiquitous in *Escherichia coli* [46], *Brucella* [47], *Yersinia pestis* [48], *Pseudomonas* [49], and *Staphylococcus aureus* [50]. Studies of Tcps in other bacteria demonstrated toxicity and allowed microbial escape from immune recognition [46,47,48,49,50]. It is worth noting that only highly virulent bacterial strains carry Tcps [46,47,48,49,50], whereas knockout strains are less virulent. Furthermore, we found that the highly virulent *Y. ruckeri* SC09 strain carried *stir-2*, whereas the non-pathogenic environmental strain *Y. ruckeri* SC10 did not harbor this gene.

Some bacterial compounds, including lipopolysaccharides and peptidoglycans, can be used as alarmins in the infection site [51]. The interaction of these signals with TLR activates immune functions in cells [21,22]. The danger signal stimulates the innate immune system of the host. Bacterial TIRs can play a competitive role in blocking signal transmission because their sequences are similar to those of intracellular TLRs [28,45,47,49]. Bacterial TIRs may block immune responses by competitively binding to the MyD88 linker protein in the NF-κB signaling pathway [47,49]. Our results showed that STIR-2 bound to MyD88 in the *Y. ruckeri* SC09 strain. In addition, in vitro experiments demonstrated the ability of STIR-2 to block the TLR2- and TLR4-mediated activation of NF-κB. NF-κB is an important transcription factor in the fish immune system and initiates the expression of inflammatory cytokines [52]. The inhibition of the NF-κB signaling pathway decreases the production of downstream inflammatory cytokines. Our results suggest that *stir-2* significantly affects NF-κB-mediated transcription and cytokine production, contributing to bacterial survival and replication in host cells. Furthermore, the present results demonstrated the intracellular viability of wild-type *Y. ruckeri* SC09 and its ability to colonize host organs.

STIR-2 as a TIR homologue is a very interesting protein. Our results demonstrated that STIR-2 was located adjacent to the type IV secretion system (T4SS) in the ICE, suggesting that STIR-2 secretion may be related to the T4SS. Studies on *Brucella* [47] found that the secretion of Tcps was mediated by the T4SS. Therefore, additional studies are necessary to elucidate the mechanisms involved in the interaction between STIR-2 and T4SS in *Y. ruckeri*.

## 4. Materials and Methods

### 4.1. *Bacterial Strains*

Wild-type *Y. ruckeri* SC09 was isolated from diseased fish in a commercial farm in Jianyang County, Sichuan province of China, and was routinely cultured in Luria-Bertani (LB) medium at 28 °C. The virulent *Y. ruckeri* SC10 strain was also isolated from the aquatic environment.

### 4.2. *Construction of Y. ruckeri ∆stir-2 Mutant and Complemented Strains*

Gene knockout was previously described by Luo et al. [53]. The *stir-2* gene sequence (gene accession number: NJ56_RS12445) of the *Y. ruckeri* SC09 strain (genome accession number: NZ_CP025800) is available in GenBank. The left and right homology arm primer sequences of *stir-2* were GGAATCTAGACCTTGAGTCGATGACTGGACGCTCGATGAAG/ ACGACTGGAAGGATGGGGATAACCGCGCAGGATTCATTAT (upstream, A) and ATAATGAATCCTGCGCGGTTATCCCCATCCTTCCAGTCGT/ACAGCTAGCGACGATATGTCGTCCTCTTGTCATTTATTCCCCA (downstream, B). The left and right homology arms AB of the *stir-2* gene were constructed, and AB was cloned into pLP12 (KnoGen Biotech Co., Ltd., Guangzhou, China) to form the pLP12-*stir-2* construct. pLP12-*stir-2* was transformed into competent the *E. coli* strain β2163 (KnoGen Biotech Co., Ltd., Guangzhou, China) by electro-transformation. A positive strain resistant to chloramphenicol was isolated and designated pLP12-*stir-2*-β2163. The co-culture of β2163 cells containing pLP12-*stir-2*-positive clones with *Y. ruckeri* SC09 resulted in conjugation and allowed screening for the first homologous recombination in the mutant SC09 strain in LB plates (20 μg/mL CM + 0.3% D-glucose). The SC09 strains with the insertion mutation were screened on LB plates (0.4% L-arabinose) to obtain a ∆*stir-2* strain with a second homologous recombination. As previously described [53], the SC09 and SC10 strains were re-transformed with the *stir-2*-pBAD33cm-rp4 vector [54], and the expression of *stir-2* gene was induced with arabinose.

### 4.3. *Fish Infection Model*

In the logarithmic growth phase, the wild-type *Y. ruckeri* SC09 and recombinant SC09Δ*stir-2* were inoculated intraperitoneally (dose: 5 × 10^7^ CFU) into 15 random rainbow trout (weight, 60–100 g), and fish death was determined. Rainbow trout survival curve analysis and mapping were performed using GraphPad Prism software (version 8.0, Dr. Harvey Motulsky, San Diego, California, USA). The growth curves of *Y. ruckeri* SC09 and SC09Δ*stir-2* were determined to rule out the effect of the difference in growth ability of the knockout strain on the infection model. To investigate infection and histological differences in immune organs after fish infection, the liver, spleen, and kidney of dead rainbow trout were homogenized under aseptic conditions, and the homogenates were diluted 10 times using plate technology. Bacterial cells were counted on a plate containing nutrient agar supplemented with triphenyl tetrazolium chloride, and the bacterial load was determined in each organ. Furthermore, the liver, kidney, and spleen of rainbow trout were harvested in mid-infection (5 days after infection) for routine paraffin embedding and HE staining.

### 4.4. Bacterial Adherence, Invasion, and Intracellular Survival Assays

Head kidney macrophages of rainbow trout were separated according to the method of Jiang et al. [55] and used to determine the effects of the *Y. ruckeri stir-2* gene on bacterial adherence, invasion, and intracellular survival. Macrophages were seeded at approximately 2 × 10^5^ cells per well in 24-well plates and grown in medium 199 (M199) (Hyclone, Shanghai, china) with 10% fetal bovine serum (FBS) (Hyclone, Shanghai, china) at 20 °C for 24 h. For the adherence assay, the cell monolayer was washed twice with M199 and infected with *Y. ruckeri* SC09, *Y. ruckeri* SC09Δ*stir-2*, or *Y. ruckeri*∆*stir-2* + pSTIR-2 at a multiplicity of infection (MOI) of 1.5. Bacteria were centrifuged onto the cells at 400× *g* for 5 min, and cells were incubated at 20 °C for 1 h. Nonadherent bacteria were removed by rinsing the wells twice with D-PBS (Hyclone, Shanghai, china). The cells were detached from the plate using 100 μL of 0.2% Triton X-100 in sterile water and 900 μL of M199. The cell suspension was serially diluted 10-fold with D-PBS and spread onto LB agar plates to determine the number of viable bacteria. For the invasion assay, the culture, infection, and counting of bacterial cells were performed as described for the bacterial adherence assay, except that extracellular bacteria were incubated, washed twice with D-PBS, and killed by incubating the monolayers with M199 containing gentamicin (100 μg/mL) for 1 h. For the bacterial intracellular survival assay, bacterial cell culture and counting were performed as described for the bacterial adherence assay. Rainbow trout head kidney macrophages were infected with either *Y. ruckeri* SC09 or *Y. ruckeri* SC09Δ*stir-2* at a MOI of 1.5 and incubated in M199 containing 3% FBS and 50 g/mL gentamicin. The cells were washed and lysed at 1, 4, 8, 12, 18, 24, 48, and 72 hpi to determine the rate of bacterial recovery. All assays were performed in sextuplicate wells, and the results are averages of at least six independent experiments. The intracellular survival rate of the cells 48 h after infection with wild-type bacteria was determined by transmission electron microscopy. To confirm that gene knockout inhibited STIR-2 protein expression in cells, we used rabbit anti-STIR-2 antibody to test the ability of knockout, wild-type, and recombinant strains to produce proteins. In addition, to prove the effectiveness of the recombinant strains, we further complemented the *stir-2* expression plasmid in the SC10 strain, which lacked *stir-2*, and used rabbit anti-STIR-2 antibody to measure protein synthesis.

### 4.5. Enzyme-Linked Immunosorbent Assays and qPCR

ELISA was performed using sets of fish (Meimian Industrial Co., Ltd., Jiangsu, China) to quantify IL-6, IL-1β, and TNF-α in culture supernatants. For qPCR, tissue homogenates and supernatants from infected cells were prepared by extracting total RNA with RNAiso Plus (Takara, Dalian, China) according to the manufacturer’s standard protocol. cDNA was synthesized from RNA using the Prime Script RT reagent Kit (TaKaRa, Dalian, China). The gene expression levels of immune-related genes (IL-6, IL-1β, and TNF-α) were analyzed by real-time PCR. The β-actin gene of rainbow trout was selected because of its lower expression variability. The following primers specific for rainbow trout were used: IL-1β-F, 5′-TGAGAACAAGTGCTGGGTCC-3′ and IL-1β-R, 5′-GGCTACAGGTCTGGCTTCAG-3′ (148 bp); IL-6-F, 5′-GAGTTTCAGAAGCCCGTGGA-3′ and IL-6-R, 5′-AGCTGGTACACTTGCAGACC-3′ (149 bp); TNF-α-F, 5′-CACACTGGGCTCTTCTTCGT and TNF-α-R, 5′-CAAACTGACCTTACCCCGCT-3′ (155 bp).

### 4.6. Construction of Prokaryotic Expression Vectors

The full-length *Y. ruckeri* SC09 *stir-2* was cloned into pET28a (Invitrogen, Shanghai, china), generating the pET28-stir-2 construct. The following primers were used: 5′-ATGATCACTATTTTGCA-3′ and 5′-ATAATGTGCGCAACCGG-3′. pET28-*stir-2* was used for producing the recombinant STIR-2 protein. The full-length *Y. ruckeri* SC09 *stir-2* was also cloned into pGEX-6P-1 (Invitrogen), generating the pGEX-*stir-2* construct, which carried the tac promoter, N-terminal GST tag, and ampicillin resistance gene. The following primers were used: 5′-CGGGATCCATGATCACTATTTTGCA-3′ and 5′-CGCTCGAGATAATGTGCGCAACCGG-3′. The pGEX-*stir-2* was used for producing the recombinant STIR-2-GST protein.

### 4.7. Construction of Eukaryotic Expression Vectors

The pCMV-HA-MyD88 plasmid was purchased from Wuhan Miaoling Bioscience & Technology Co., Ltd. STIR-2 was cloned into the eukaryotic expression plasmids pCMV-Flag-2B (Stratagene, Beijing, china), and the plasmid pSTIR-2-Flag was produced using the following PCR primers: 5′-ACCATGGATTACAAGGATGAATGATCACTATTTTGCA-3′ and 5′-GGGCCCCCCCTCGAGGTCGAATAATGTGCGCAACCGG-3′.

### 4.8. Transfection and NF-κB-Dependent Luciferase Reporter Assay

Rainbow trout head kidney macrophages were transiently transfected for 12 h using Lipofectamine™ 3000 Transfection Reagent (Life, Shanghai, china), according to the manufacturer’s instructions, with 0.3 μg of DNA, including 50 ng of TLR-4 and TLR-2 plasmids (Miaoling Bioscience & Technology Co., Ltd., Wuhan, China), 200 ng of pBIIXLuc reporter plasmid, and 50 ng of the FLAG-*stir-2* expression vector. The total amount of DNA was kept constant by adding empty vector. Where indicated, cells were treated with *E. coli* LPS (Invivogen) and Pam2CSK4 (Invivogen) for 8 h and lysed. Luciferase activity was measured using the Dual-Glo^®^ Luciferase Assay System (Promega, Beijing, china). The *stir-2* sequences from SC09 genomic DNA were ligated into pCMV-Tag 2B using Exnase II (ClonExpress II, Vazyme, Nanjing, china).

### 4.9. Co-Immunoprecipitations (CO-IP)

Rainbow trout head kidney macrophages were infected with wild-type SC09 for 12 h. Cells were washed twice in ice-cold PBS, harvested, and cell lysate was added. Cell lysis and processing for co-immunoprecipitation were performed using the Pierce™ Co-Immunoprecipitation Kit (Thermo Scientific, Shanghai, china). The eluted IP samples were detected by western blotting using rabbit anti-STIR-2 (prepared in this study) or anti-MyD88 (Abcam, Shanghai, china) antibody.

### 4.10. Pulldowns from Cell Extracts

Macrophages were transiently transfected with the pCMV-HA-MyD88 plasmid using Lipofectamine™ 3000 Transfection Reagent (Life). Eighteen hours after infection, cells were washed in ice-cold PBS, harvested, and resuspended in RIPA buffer (Sigma-Aldrich, Shanghai, china). Extracts were centrifuged at 16,000× *g* at 4 °C for 20 min. The supernatant was incubated with the recombinant STIR-2-GST protein at 4 °C for 3 h, loaded into a gravity flow column containing glutathione-Sepharose (GE, Shanghai, china) for 1 h, washed in water, and equilibrated in equilibration buffer (20 mM Tris–HCl,). The column was washed twice in equilibration buffer, and proteins were eluted with 1% Triton PBS. The eluted proteins were separated by SDS-PAGE, transferred to a PVDF membrane, incubated with anti-HA (Abcam), anti-MyD88 (Abcam), or anti-STIR-2 antibody for 50 min, and detected with horseradish peroxidase (HRP)-conjugated secondary antibody.

### 4.11. Yeast Two-Hybrid Assay

The plasmids used in the two-hybrid system were constructed using Exnase II (ClonExpress II, Vazyme, Vazyme). *myD88* and *Y. ruckeri* SC09 *stir-2* were amplified from the pCMV-HA-MyD88 vector and SC09 genomic DNA, respectively, by PCR using Exnase II primers. PCR products were cloned into the pGBKT7 vector downstream of the Gal4 DNA-binding domain (BD) (Clontech, Shanghai, china) containing the screening marker gene *trp* and into the pGADT7 vector downstream of the Gal4 activation domain (AD) containing the marker gene *leu*. Y2HGold yeast competent cells were transformed with BD and AD fusion protein vectors. Diploid yeast cells carrying both plasmids were obtained by mating and selected in synthetic dextrose medium (SD) lacking leucine (*leu*) and tryptophan (*trp*). Protein interactions were assessed in medium lacking histidine (*his*). The β-galactosidase expression filter assay was performed using the LacZ reporter gene. The primers used in the two-hybrid system were GWMyD88F-5′-CATATGGCCATGGAGGCCGAGCTGCAGGAGGTCCCGGCGC-3′, 5′-GWMyD88R-GCGGCCGCTGCAGGTCGACGTCAGGGCAGGGACAAGGCCT-3′; 5′-GW*stir-2*F-CATATGGCCATGGAGGCCAGATGATCACTATTTTGCA-3′, 5′-GW*stir-2*R-CTGCAGCTCGAGCTCGATGGATAATGTGCGCAACCGG-3′.
